# Being Bullied in Virtual Environments: Experiences and Reactions of Male and Female Students to a Male or Female Oppressor

**DOI:** 10.3389/fpsyg.2018.00253

**Published:** 2018-03-06

**Authors:** Nicole Krämer, Sabrina Sobieraj, Dan Feng, Elisabeth Trubina, Stacy Marsella

**Affiliations:** ^1^Computer Science and Applied Cognitive Science, University of Duisburg-Essen, Essen, Germany; ^2^College of Computer and Information Science, Northeastern University, Boston, MA, United States

**Keywords:** virtual environments, bullying, gender, psychophysiology, resilience, psychological

## Abstract

Bullying is a pressing societal problem. As such, it is important to gain a better understanding of the mechanisms involved in bullying and of resilience factors which might protect victims. Moreover, it is necessary to provide tools that can train potential victims to strengthen their resilience. To facilitate both of these goals, the current study tests a recently developed virtual environment that puts participants in the role of a victim who is being oppressed by a superior. In a 2 × 2 between-subjects experiment (*N* = 81), we measured the effects of gender of the oppressor and gender of the participant on psychophysiological reactions, subjective experiences and willingness to report the event. The results reveal that even when a male and a female bully show the exact same behavior, the male bully is perceived as more threatening. In terms of gender of the victim, the only difference that emerged was a more pronounced increase in heart rate in males. The results were moderated by the personality factors social gender, neuroticism, and need to belong, while self-esteem did not show any moderating influence.

## Introduction

The use of virtual environments (VE) is nowadays widespread. Their potential in academia has been discussed extensively, and numerous research applications have been presented. Since VEs offer the possibility to create settings of high ecological validity that can be fully controlled, they have been suggested for and employed in fundamental research (Blascovich et al., 2002; Mapala et al., 2017) and for therapeutic and training purposes (e.g., Bossard et al., 2007; Potkonjak et al., 2016). Fundamental research uses virtual environments to study and understand fundamental mechanisms, for example regarding deceptive behavior (Mapala et al., 2017) or proxemics behavior (Yee et al., 2007; Iachini et al., 2016). Moreover, VEs can be employed to examine and reduce stereotype bias in terms of racial or age stereotypes (Banakou et al., 2016; Oh et al., 2016; Hasler et al., 2017). In the applied area of therapeutic interventions, virtual scenarios are being tested for the treatment of paranoia, post-traumatic stress disorders and other anxiety disorders (Gerardi et al., 2010) such as flight anxiety (Cardoş et al., 2017) and speech anxiety (Pertaub et al., 2002). Increasingly, they are also being used for training purposes, mainly in the area of training motor skills, for example regarding surgery (Seymour et al., 2002), motor rehabilitation training (Holden, 2005; Pedreira da Fonseca et al., 2017) or to perfect skills in sports (Miles et al., 2012).

The development and evaluation of virtual environments for training resilience and future behavior in stressful situations has not been extensively addressed. Most notable among the exceptions is the stress resilience training conducted with military service members prior to their initial deployment (Rizzo et al., 2013). Here, users are immersed in a challenging context and train a range of psychoeducational and cognitive-behavioral emotional coping strategies believed to enhance stress resilience. More recently, a virtual environment application has been presented that enables resilience training for bullying situations (Feng et al., 2017; Jeong et al., 2017). Given its societal importance, bullying represents a critical, and yet widely neglected, field of application for virtual environment resilience training. Bullying can occur in various forms: as physical (e.g., slapping), verbal (e.g., offensive utterances), and relational (e.g., betrayal, social exclusion, spreading harmful gossip) violence (Berger, 2007). Due to its high prevalence rates in the population, bullying can be seen as a pressing societal problem. Indeed, in a meta-analytic review, Berger (2007) estimated that approximately 9–25% of school children worldwide have already been victims of bullying.

Bullying can have a powerful impact on the victims, in terms of negative affect (e.g., feeling nervous) and physiological reactions (e.g., stress, headaches, pain, sleep problems) (Hansen et al., 2006). It can also cause long-term consequences such as depression (Agervold and Mikkelsen, 2004; Sapouna and Wolke, 2013).

In the current paper, we test the recently developed virtual environment application (Feng et al., 2017) with a special focus on the influence of gender (of the bully as well as the victim) and personality factors. The aim is twofold: We (a) employ virtual environment technology as an empirical testbed in order to learn more about the mechanisms and resilience factors influencing the effects of bullying (specifically regarding the impact of the bully’s gender and the participant’s personality variables) and (b) evaluate the effects of the environment on different groups of participants with regard to their stress levels, emotional states and behavioral intentions. The results of these analyses should form the basis for an effective training intervention which could be applied to train victims or enhance prevention workshops in schools or universities.

## Theoretical Background

### Virtual Environments

Virtual environments are synthetic replications of the real world or of specific situations. Users are provided with the experience of being surrounded by these environments (Loomis et al., 1999), and they are often perceived as real. To immerse and interact in the environment, “[u]sers wear displays that fully immerse a number of the senses in computer generated stimuli. Stereoscopic head-mounted displays (HMD) are a distinctive feature of such systems” (Biocca and Delaney, 1995, p. 56). Virtual environments offer the possibility to vary characteristics of situations in very subtle ways: Environmental cues (e.g., creating a classroom, a farm or anything else) and social cues (e.g., the number of virtual persons present, their gender) can be systematically manipulated in order to examine their influence on participants’ social interaction, cognition and behavior (Blascovich et al., 2002; Bombari et al., 2015; Maister et al., 2015). One essential advantage of virtual environments lies in their possibility to enable persons to test their responses under fairly realistic conditions, without serious consequences. Therefore, virtual environments are nowadays successfully employed in a variety of settings for research and educational or training purposes. For instance, they are used for disaster training for healthcare professionals (Farra et al., 2015), police personnel (Bertram et al., 2015) or even for civilians learning how to behave in the case of an unexpected fire emergency (Gamberini et al., 2003). Moreover, they are implemented to treat paranoia, post-traumatic stress disorders, and other anxiety disorders (Slater et al., 2006; Gerardi et al., 2010; Atherton et al., 2016; Cardoş et al., 2017). In addition, virtual environments are used for fundamental research in order to understand basic mechanisms, for instance, as mentioned above, regarding proxemics behavior, deception, or stereotype bias. Given these applications, it therefore seems feasible to employ a virtual scenario that can (a) serve highly controlled experimental research on the mechanisms and influencing factors underlying victims’ responses and potential resilience and (b) be refined to serve as an environment in which to train appropriate reactions and resilience. In order to employ virtual environments for both fundamental and applied research goals, it is necessary to demonstrate that the environment is able to elicit emotional and psychophysiological responses. Previous research demonstrated that virtual environments can indeed elicit strong emotional reactions (Slater et al., 2006; Pan and Slater, 2011). Moreover, Kotlyar et al. (2008) found that both blood pressure and heart rate were significantly increased in response to a speech stressor presented in a virtual environment. Most recently, Kothgassner et al. (2016) found comparable physiological stress responses in participants undergoing a public speaking task in a real-audience and a virtual-audience condition.

In conclusion, most findings indicate that virtual environments induce similar emotional and physiological reactions to those elicited in real-life situations, and compared to classic training methods (e.g., Pan and Slater, 2011; Kothgassner et al., 2016). We therefore suggest that a virtual scenario can be employed in a (mild) bullying setting to examine victims’ reactions by measuring physiological (during) and emotional reactions (afterward). In this way, we aim to contribute evidence regarding the influencing factors for emotional reactions and resilience. We further aim to derive suggestions for refining the environment for applied settings such as resilience training interventions.

### Research on Bullying

Juvonen and Graham (2014) state that “[b]ullying involves targeted intimidation or humiliation. Typically, a physically stronger or socially more prominent person (ab)uses her/his power to threaten, demean, or belittle another. To make the target or victim feel powerless, (…)” (p. 161). While some researchers believe that bullying has to occur on a regular basis to have adverse effects (Olweus, 1993), Juvonen and Graham (2014) suggest that even one single mistreatment can be sufficient to elicit fear of further bullying. The negative consequences can range from negative feelings to severe psychophysiological reactions and clinical depression (Hansen et al., 2006). Bullying can be seen as a stress event, as described by Selye (1950) or Lazarus and Folkman (1984).

One key characteristic of bullying is the power imbalance between the involved parties; there is always a bully (or perpetrator) and a victim. Wolke and Skew (2012) report that the roles (victims, bullies) are remarkably stable over time. This is underlined by recent meta-analytic findings of Kljakovic and Hunt (2016), who confirmed the stability of roles with a large effect size. Einarsen (1999) reported that personality traits of the victim and psychosocial factors are decisive regarding the question of who becomes a victim.

#### Prevalence of Bullying

Bullying is a societal problem which affects children, adolescents and adults. Referring to German, Austrian and English studies, Einarsen (1999) speculates that 70–80% of working adults have been bullied by their supervisors. For United States workers, Lutgen-Sandvik et al. (2007) estimated that 35–50% have been affected. Other data suggest that only approximately 10–25% of the adult population across different countries (e.g., Europe, United States) has been affected by bullying (Wolke and Skew, 2012; van Heugten, 2013). Although a wide range of persons are affected by bullying, and the consequences can be devastating, some victims have the resources to cope with the difficult situation and to adjust in a positive way; they seem to be resilient. Research on resilience is currently focusing on the complex interplay of social resources (outside the family), family support and personal characteristics (Sapouna and Wolke, 2013). However, although resilience is an important factor, it has not received a great deal of attention in this context. This might be due to the difficulty of investigating resilience through survey studies, which are widely used in bullying research. As self-reports can become distorted over time, especially concerning felt emotions and immediate reactions, it is hard to identify the relation between personal resources and immediate reactions to bullying. Thus, one goal of the current study is to focus on resilience factors inherent in the victim and to relate these to the reactions that occur in the bullying situation.

#### Gender Differences in Bullying

There is consistent evidence that boys and male adolescents more often act as bullies compared to their female counterparts (Barlett and Coyne, 2014; Narayanan and Betts, 2014), especially with respect to physical bullying (Juvonen and Graham, 2014).

Moreover, studies have also demonstrated that boys and male adolescents are more frequently the victims of bullying (Wolke and Skew, 2012; Narayanan and Betts, 2014) compared to females. However, other studies found no gender effect (Kljakovic and Hunt, 2016), or that females were more likely to become victims of relational bullying (Sapouna and Wolke, 2013).

With regard to gender differences in the potential consequences of being bullied, and the question of whether gender might also function as a protective factor in terms of resilience, Sapouna and Wolke (2013) reported that males tend to show lower levels of depression, while females tend to be more vulnerable to depression. This was also demonstrated by Turner et al. (2013) with regard to cyberbullying. With regard to coping behavior, compared to men, women seem to be more willing to (a) to report their mistreatment to authorities and friends and (b) seek help (Unnever and Cornell, 2004; Kostev et al., 2013). Unnever and Cornell (2004) reported that girls found it easier to talk to their friends about victimization than to adults, who might rather be perceived as authorities. Approximately 30 percent of students do not report their victimization at all, because they are scared and do not believe that authorities in particular would be able to change their situation (Unnever and Cornell, 2004; Berger, 2007). Berger (2007) reported a positive effect of talking with peers about mistreatment. In line with these findings and open questions, the present study aims to evaluate whether a virtual bullying situation can be used as a testbed to learn about the factors influencing the willingness to report bullying.

While the aforementioned findings relate to biological gender, the literature also indicates that social gender can be a further determining factor. Social gender refers to personal characteristics, and addresses whether an individual has rather female or male attributes. Attributes that are perceived as female are communality, warmth and expressivity, while supposedly male attributes include instrumentality and dominance. People with atypical characteristics have been shown to be victimized more often (Navarro et al., 2015). Thus, it seems that both the biological and the social gender can predict involvement in a bullying situation. To our knowledge, the potential for resilience with respect to social gender has not yet been addressed, although it has been suggested as one of the personality traits influencing adjustment after victimization (e.g., Donnon and Hammond, 2007). Nevertheless, a person’s social gender attributes might predict adverse reactions to a greater degree than biological attributes. For instance, oppression might induce more aversion in a person who is sensitive, sociable and caring (female attributes, Prentice and Carranza, 2002) than in a person who is assertive, competitive and aggressive (male attributes, Prentice and Carranza, 2002).

##### Impact of bullies depending on their gender

Another unanswered question refers to the impact of the bully depending on his/her biological gender. According to gender stereotypes, men can be perceived as more threatening; thus, it can be asked whether male and female perpetrators are perceived in the same way. Men are seen as agentic and holding attributes like assertiveness and aggression, while women are associated with warmth and communality (see Cuddy et al., 2008). Additionally, men commonly have different physical attributes, which might be perceived as more menacing. On the other hand, female bullies might be perceived as more threatening because counter-stereotypical behaviors (i.e., being suppressive, dominant and aggressive instead of warm and kind) are unexpected and can lead to penalization (e.g., Eagly and Karau, 2002; Bosson and Michniewicz, 2013).

#### Individual Differences in Bullying

As outlined above, it has repeatedly been suggested that it is not random who gets involved in bullying situations (Juvonen and Graham, 2014). As such, it has been discussed whether personality factors are associated with victimization (Einarsen et al., 1994). For example, studies demonstrated that victimization was positively correlated with neuroticism and negatively correlated with conscientiousness (Bollmer et al., 2006; Zapf and Einarsen, 2011; Wolke and Skew, 2012; Kodžopeljić et al., 2014; Nielsen and Knardahl, 2015). Zapf and Einarsen (2011) summarized that while some studies found extroversion, agreeableness and conscientiousness to be associated with victimization, others did not. Additionally, self-esteem and self-assertiveness can be important factors (Zapf and Einarsen, 2011). For instance, Baumeister et al. (2003) found that persons with low self-esteem ratings were more often victims than persons with high self-esteem. In addition, Zapf and Einarsen (2011) reported that victims score high on sensitivity, suspiciousness, anxiety and depression and low on assertiveness and competitiveness.

Individual traits also play a decisive role in terms of resilience (Sapouna and Wolke, 2013). In a more general context not specifically related to bullying, Friborg et al. (2005) stated that resilience was related to “high score[s] on emotional stability [low neuroticism], extroversion, openness and conscientiousness […], as well as agreeableness…”. They found a strong negative correlation between neuroticism and resilience, and revealed that neurotic persons stated more negative affect and showed more symptoms of anxiety and depression. Transferring these results to resilience against bullying, it can be expected that victims with low scores on neuroticism will report less negative reactions (e.g., negative affect) than victims with high neuroticism scores.

Sapouna and Wolke (2013) found that high self-esteem is positively associated with positive adjustment after victimization. Von Soest et al. (2010) further suggested that hardiness and a positive cognition of events (e.g., seeing chances/opportunities in negative situations/experiences) can lead to less negative reactions to stressful experiences. Van Heugten (2013) added that the perceived level of control on the part of the victim has an impact on the outcome of the bullying situation. In line with Sapouna and Wolke (2013), we therefore suggest that self-esteem is positively associated with resilience and less negative reactions to victimization.

Another moderating factor might be the “need to belong, that is, a need to form and maintain at least a minimum quantity of interpersonal relationships (…)” (Baumeister and Leary, 1995, p. 499). While it is fairly well testified that bullies strive for acceptance from their peers (e.g., Olthof and Goossens, 2008), the role of the need to belong on the part of the victim has received less research attention. For victims, the need to belong might especially affect the willingness to approach others after a bullying event.

To sum up, a broad body of research has found that bullying leads to stress reactions in terms of negative affect (e.g., feeling nervous) and physiological reactions (e.g., increased electrodermal activity). Moreover, (personality) traits of the victim (e.g., high neuroticism scores, gender) as well as attributes of the bully (e.g., male competitors are perceived as more dominant) seem to be influential. Although it is well known who is affected by bullying, less is known about resilience factors inherent in the victims. Most researchers applied survey studies to gain insights into bullying processes. While such studies provided a great deal of valuable results, the exploratory power of these results is partly limited. As virtual environments offer the opportunity to create situations of high control and systematization (Loomis et al., 1999; Blascovich et al., 2002), we strive to employ a virtual scenario in order to extend the basic research on these issues.

#### Research Questions and Hypotheses

Studies have revealed that prevalence rates of bullying are rather high, with approximately one third of the population across nations and across all ages having already been involved in bullying. The consequences can be far-reaching, especially for victims. For the experimental setting here, we specifically focus on bullying by an authority in an institutional setting, in order to represent a situation of clear power imbalance (Juvonen and Graham, 2014). Moreover, we are especially interested in the question of under which conditions bullying authorities would be reported. Regarding resilience factors, not every victim is permanently hurt/psychologically impaired by bullying; some victims show positive adjustments due to their coping potential (Lazarus and Folkman, 1984). Referring to the current literature on bullying and resilience (see Individual Differences in Bullying; Friborg et al., 2005; Sapouna and Wolke, 2013), we assume different personality attributes to be important, such as neuroticism, self-esteem and need to belong. Furthermore, the biological and social gender have been assumed to influence the victim’s reaction to bullying (Prentice and Carranza, 2002; Turner et al., 2013). Based on this previous work, we state the following hypotheses:

H1: Female victims will experience more adverse reactions (based on self-reports and physiological reactions) than male victims. These reactions will be moderated by the victim’s personal characteristics (social gender, neuroticism, self-esteem and need to belong).

According to gender stereotypes, we assume that the biological gender of the bully can be an influencing factor.

H2: A male bully will elicit more adverse reactions (based on self-reports and physiological reactions) than a female bully. These reactions will be moderated by the victim’s personal characteristics (social gender, neuroticism, self-esteem and need to belong).

Moreover, we suppose an interaction between the biological sex of the victim and the biological sex of the bully.

H3: Female victims oppressed by a male bully will show more adverse reactions (based on self-reports and physiological reactions) than male victims oppressed by a male bully, female victims oppressed by a female bully, and male victims oppressed by a female bully. These reactions will be moderated by the victim’s personal characteristics (social gender, neuroticism, self-esteem and need to belong).

## Materials and Methods

### Study Design and Virtual Scenario

**FIGURE 1:**
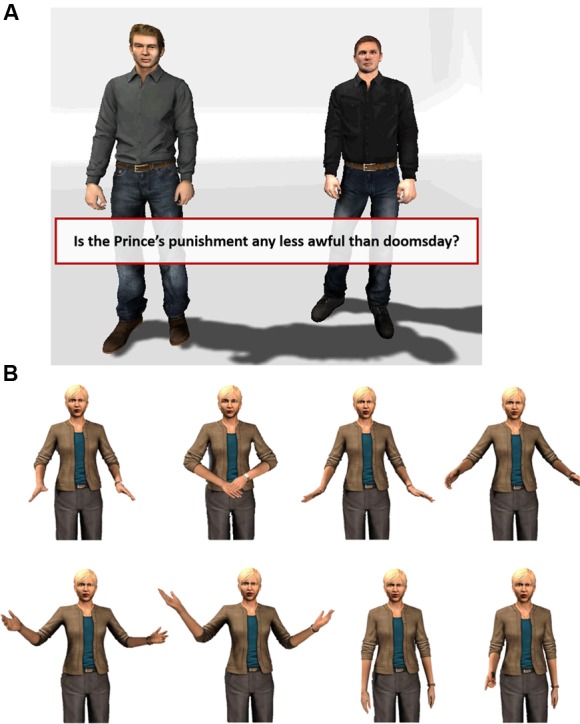
**(A)** Screenshot of the virtual environment. The character standing on the left is the virtual instructor and the character on the right is the virtual fellow student. The participant’s lines appear on the 3D interface in front of the participant. **(B)** The example of negative non-verbal behaviors when the virtual instructor said “No, that’s not right. Honestly, how hard is it? Do it again!”

To examine our hypotheses, we conducted an experiment with a 2 (bully’s gender) × 2 (victim’s gender) between-subject design (*N* = 81, 45 females, 36 males). The virtual environment was designed to simulate a bullying scenario by assigning the participants to a task that is impossible to complete to the bully’s satisfaction. As shown in **Figure 1A**, the virtual environment is a wide open space in which two virtual characters are displayed. Specifically, we simulated a rehearsal in an acting class scenario, because this is a situation in which feedback can be given naturally. To create the imbalance of power, the key element of bullying behavior, one of the virtual characters was designed to be the instructor (female/male) and the authority in the scene. The participants took the role of an acting student, reading lines from a script and interacting with a second (virtual) student who was also practicing his lines while taking instructions from a virtual instructor. The participant could see both the instructor and the fellow student (**Figure 1A**) standing in a neutral, stage-like room. The virtual fellow student served to simulate a real-life acting class in which participants rehearse a scene together, as well as to enhance the participants’ feeling of being treated differently. The fellow student looked the same in all conditions and displayed the same, neutral behavior, saying his lines with default gaze behaviors following the person who speaks. Participants were asked to rehearse the script adapted from ‘Romeo and Juliet: Act 3, Scene 3,’ with the virtual fellow student playing another character in the script, Friar Lawrence, while the participant played Romeo. There were no other interactions between the participant and the virtual student, beyond reading their different parts in the script. The researcher told the participants that their goal was to finish their rehearsal in a limited amount of time. Each time the participant finished reading a line, the virtual instructor provided feedback. Participants were told that the instructor’s feedback was specifically tailored to their performance, and that they should follow the instructor’s directions to the best of their ability. The negative feedback from the virtual instructor was scripted and identical for all participants regardless of their performance. Each time the participant finished reading a line, the virtual instructor verbally bullied the participants by providing strong negative feedback, using harsh language and even ridicule, with negative non-verbal behaviors (see **Figure 1B**). For example, the negative feedback included sentences such as “Ugh, stop. You sound like a dead fish,” “No no no, that’s not right. Honestly, how hard is it?” and “Come on, work with me here. Say it like you mean it.” (For a video of the situation featuring the male perpetrator see **Supplementary Materials**. Please note that the participants watched this with an oculus, i.e., saw only one picture).

#### System Apparatus

**FIGURE 2:**
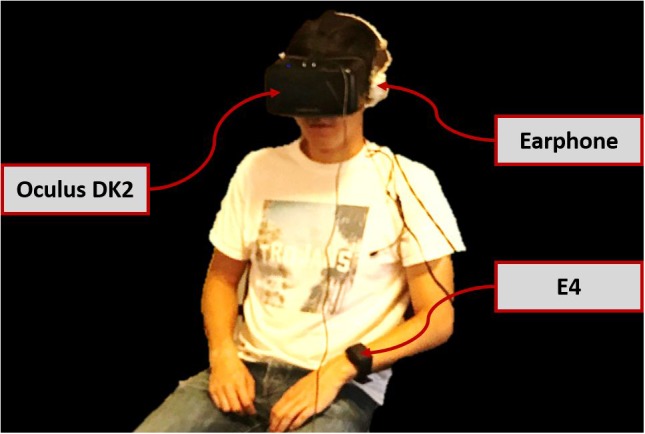
System apparatus.

The 3D virtual environment was developed using Unity3D. The virtual humans’ non-verbal behaviors such as facial expression and gestures were automatically generated using Cerebella (Lhommet and Marsella, 2013; Marsella et al., 2013) and the generated animations were controlled using Virtual Human Toolkit. The head-mounted display (HMD) was the Oculus Rift Development Kit 2. The experiment apparatus is shown in **Figure 2**. An Empatica E4 sensor measured physiological signals, heart rate and electrodermal activity.

### Procedure

The experiment took place in a virtual reality lab at the University Duisburg-Essen, Germany. Participants were recruited by personal contact online and offline. When they arrived at the university, they were welcomed by the experimenter, instructed about the setting and asked to provide informed consent. Then, the participants took a seat and were fitted with the Empatica E4 and asked to fill in the first part of the questionnaire including the personality traits. Afterward, the experimenter fitted the participants with the Oculus Rift. The experimenter started (a) the recording of the physiological data by tagging the Empatica E4 and (b) the video recording, and ended the recording after the interaction. Interactions took about three to 4 min; the total duration of the experiment was approximately 30 min. Finally, participants filled in the second part of the questionnaire and were debriefed.

### Measures

To examine our hypotheses, we captured personality traits and participants’ adverse reactions. In addition, participants’ sociodemographic characteristics were determined (biological gender, age, level of education).

#### Personality Traits

We used a subscale of the NEO-FFI (Borkenau and Ostendorf, 2008) to measure neuroticism (α = 0.810). Ratings were given on 5-point Likert scales (0 = not at all; 4 = absolutely), with high scores indicating a strong manifestation of the trait.

To measure the social gender, we employed the German version of the Personal Attributes Questionnaire (GEPAQ) by Runge et al. (1981), which comprises three subscales (eight items each): *masculinity* (M+; α = 0.632), *femininity* (F+; α = 0.663) and *masculinity-femininity* (M-F; α = 0.590). The masculinity scale includes items like *competitive* and *self-confident*, while the femininity scale includes items like *sensitive* and *emotional*. Participants gave ratings on 5-point Likert scales (0 = not at all; 4 = absolutely). For the present analyses, we only used the masculinity and femininity subscale.

Self-esteem was captured using the Rosenberg Self-Esteem Scale (Rosenberg, 1965), which consists of ten items (α = 0.838). Ratings were made on 4-point Likert scales (0 = not at all; 3 = absolutely). High values of the sum score represent high self-esteem.

We measured the need to belong with the 10-item Need to Belong Scale (Leary et al., 2013). Ratings were made on 5-point Likert scales (1 = not at all; 5 = extremely), with high scores indicating a high need to belong (α = 0.840). We additionally measured causal attribution style (McAuley et al., 1992), although this is not relevant for the present article.

#### Adverse Reactions

To examine adverse reactions, we used self-reports and physiological measures.

##### Physiological reactions

We captured electrodermal activity (EDA) and heart rate (HR) as indicators of physiological stress responses during the acting task, using the Empatica E4. This is a bracelet with four sensors (photoplethysmography, electrodermal activity sensor, accelerometer, thermometer), which can measure physiological responses in real time. Only the data of the first two sensors were used in order to derive heart rate and electrodermal activity (skin conductance level, SCL). A tagging button was used to mark the start and end of the experimental interaction. We captured a baseline before the beginning of the interaction for 3–4 min while participants were able to look around the room. For further analyses, we calculated the differences between the physiological values at baseline and at the end of the interaction to obtain values for changes in heart rate and skin conductance level.

##### Self-reports

The current mental state was measured by 28 three-point semantic differentials (Zerssen and Koeller, 1976; 0 = positive pole, 1 = indifferent; 2 = negative pole) such as *fresh-faint*, *irritated-placid* or *happy-upset*. From these, the sum score was formed, with high scores representing mental unease and low scores representing high mental well-being.

Moreover, we asked participants about their perception of the bullying situation using 16 items rated on 9-point Likert scales (1 = totally disagree; 9 = totally agree). Example items are “I felt oppressed by the instructor’s behavior” and “The instructor’s behavior made me insecure.” A factor analysis using Horn’s (1965) parallel analysis method resulted in a one-factor solution (α = 0.900); five items had to be removed from the analysis. High scores indicate a strong feeling of oppression.

##### Behavioral intentions

Finally, we measured behavioral intentions to report the mistreatment using 9-point Likert scales (1 = not at all; 9 = absolutely) after the bullying situation. We captured two different types of report: formal report (one item, “Would you report the behavior of the instructor to the university?”) and informal report (three items “Would you report the behavior of the instructor to your friends/family/fellow students?”; α = 0.846).

### Sample

Of the 83 participants who took part in the study, two participants had to be excluded (one due to technical problems and one who switched off the Empathica E4). The final study sample thus comprised 81 participants (45 females, 36 males), with an age range from 18 to 31 years (*M* = 22.70, *SD* = 2.93). As the highest level of education, approximately 94% had completed university entrance-level examinations or a higher educational qualification; the remaining 6% named another qualification (e.g., graduated from a medium-track school). On average, the participants had 9 years of experience with video games (*M* = 9.63; *SD* = 6.81). The participants in the two conditions (female/male oppressor) did not differ concerning their video-gaming experience (female oppressor condition: *M* = 10.08, *SD* = 7.46; male oppressor condition: *M* = 9.20, *SD* = 6.16). However, they differed slightly regarding their average age, with those in the female condition being 1 year older (*M* = 23.40, *SD* = 0.49) than those in the male condition (*M* = 22.02, *SD* = 0.41). Gender was distributed equally across conditions (female participants: *n*_male bully_ = 22, *n*_female bully_ = 23; male participants: *n*_male bully_ = 19, *n*_female bully_ = 17).

## Results

To examine H1–H3, we conducted a MANOVA with the independent factors bully’s gender and participants’ gender and the dependent variables physiological reactions (electrodermal activity, heart rate), self-reports (bullying perception, mental state) and behavioral intentions to report the misbehavior of the instructor (informal, formal).

Regarding H1, which stated that female participants would experience more adverse reactions, the analysis showed a difference in heart rate between female and male participants, *F*(1,77) = 5.01, *p* = 0.028, $\eta_{p}^{2}$ = 0.061: Males showed higher heart rate changes (*M* = 30.29, *SE* = 2.75, *CI* [24.82, 35.76]) than females (*M* = 22.05, *SE* = 2.45, *CI* [17.16, 26.93]). There were no significant effects on SCL [*F*(1,77) = 1.64, *p* = 0.205, $\eta_{p}^{2}$ = 0.021]. The self-reports on mental state, *F*(1,77) = 0.38, *p* = 0.542, $\eta_{p}^{2}$ = 0.005, and the perception of the bullying situation, *F*(1,77) = 0.05, *p* = 0.831, $\eta_{p}^{2}$ = 0.001, did not differ significantly. Moreover, there was no difference between men and women in the intentions to report the bullying situation in a formal, *F*(1,77) = 0.15, *p* = 0.698, $\eta_{p}^{2}$ = 0.002, or informal way, *F*(1,77) = 0.00, *p* = 0.956, $\eta_{p}^{2}$ = 0.000.

Concerning H2, which stated that a male bully would elicit more adverse effects than a female bully, the analysis revealed no significant difference in the physiological reactions depending on the bully’s gender [SCL *F*(1,77) = 2.34, *p* = 0.130, $\eta_{p}^{2}$ = 0.030; HR *F*(1,77) = 1.93, *p* = 0.169, $\eta_{p}^{2}$ = 0.024]. The self-report did not reveal a difference for the variable “mental state ratings,” *F*(1,77) = 0.15, *p* = 0.705, $\eta_{p}^{2}$ = 0.002, but a difference was found for the variable “perception of the bullying situation” depending on the bully’s gender, *F*(1,77) = 5.08, *p* = 0.027, $\eta_{p}^{2}$ = 0.062. The male bully elicited a greater threat perception (*M* = 5.52, *SE* = 0.25, *CI* [5.03, 6.02]) than did the female bully (*M* = 4.72, *SE* = 0.25, *CI* [4.22, 5.23]). Regarding the behavioral intentions to report the bullying, the analysis did not reveal a difference depending on the bully’s gender [informal *F*(1,77) = 0.14, *p* = 0.707, $\eta_{p}^{2}$ = 0.002; formal *F*(1,77) = 0.63, *p* = 0.432, $\eta_{p}^{2}$ = 0.008].

The interaction of bully’s gender and participants’ gender (H3) did not show significant differences for physiological reactions [SCL *F*(1,77) = 0.01, *p* = 0.908, $\eta_{p}^{2}$ = 0.000; HR *F*(1,77) = 0.00, *p* = 0.974, $\eta_{p}^{2}$ = 0.000], self-reports [mental state *F*(1,77) = 0.37, *p* = 0.546, $\eta_{p}^{2}$ = 0.005; perception of bullying situation *F*(1,77) = 0.45, *p* = 0.505, $\eta_{p}^{2}$ = 0.006] and behavioral intentions to report the mistreatment by the instructor [informal *F*(1,77) = 2.56, *p* = 0.114, $\eta_{p}^{2}$ = 0.032; formal *F*(1,77) = 0.70, *p* = 0.406, $\eta_{p}^{2}$ = 0.009].

To examine whether the personality variables self-esteem, need to belong, neuroticism, and social gender moderate the results of H1–H3, we conducted three-way moderations (model 3) using the PROCESS macro by Hayes (2013). To this end, we consecutively conducted moderations, with each personality trait (self-esteem, need to belong, neuroticism, and social gender) as a moderator and the physiological reactions [electrodermal activity (EDA) and heart rate], self-reports on experiences (perception of the bullying, mental state), and behavioral intentions to report the bullying behavior on a formal and informal level as dependent reactions.

### Self-Esteem

When self-esteem was used as a moderator for the relation of bully’s and participants’ gender, there was no effect of self-esteem on the dependent variables and the inclusion of self-esteem did not change any of the results depicted above.

### Need to Belong

**FIGURE 3:**
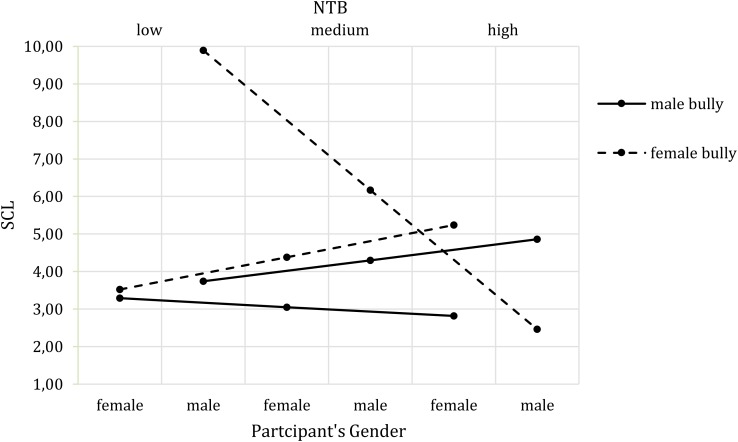
Three-way interaction effect of Bully’s Gender^∗^Participants’ Gender^∗^NTB on SCL.

In a next step, we ran analyses with need to belong (NTB) as a moderator. The overall model for skin conductance level did not reach significance *F*(7,73) = 1.51, *p* = 0.177, *R*^2^ = 0.15, but there was a significant three-way interaction effect of bully’s gender, participants’ gender and NTB on SCL (*b* = -0.80, *t*(73) = -2.51, *p* = 0.014, *CI* [-1.43, -0.16]). **Figure 3** depicts the interaction effect for low, medium and high levels of NTB. The Johnson-Neyman technique further showed that the interaction effect of bully’s gender and participants’ gender on SCL changed significantly at NTB values below -7.76 (8.64%) and above 10.49 (7.41%). The female bully elicited higher SCL in female participants with a high NTB than did the male bully, while the opposite was the case for male participants with a high NTB. Moreover, the male and female bully elicited the same SCL for female participants with a low NTB; however, male participants with a low NTB showed increased SCL in response to the female bully.

Concerning heart rate, the analysis revealed a non-significant overall model *F*(7,73) = 1.84, *p* = 0.093, *R*^2^ = 0.15. However, as in H1, there was a significant main effect of participants’ gender on heart rate, and a three-way interaction of bully’s gender, participants’ gender and NTB, (*b* = 2.41, *t*(73) = 2.05, *p* = 0.044, *CI* [0.67, 4.75]). The latter finding, however, does not show significant transition points within the moderator scores using the Johnson-Neyman technique and will therefore not be interpreted.

**FIGURE 4:**
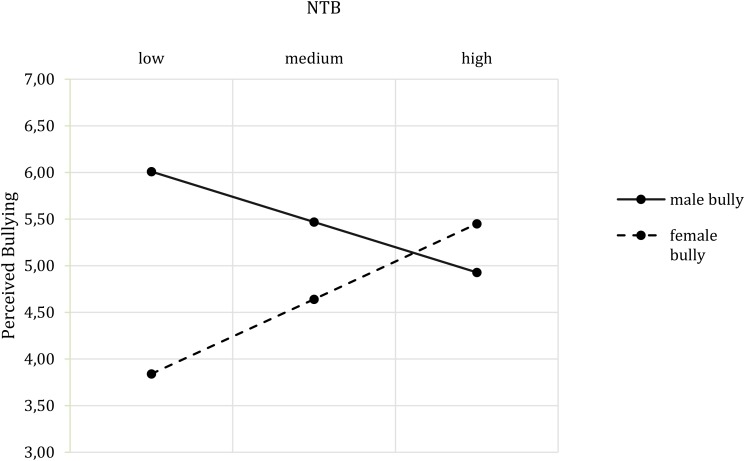
Two-way interaction effect of Bully’s Gender^∗^ NTB on perceived bullying.

With respect to the self-report data, the overall model for the perception of bullying was significant, [*F*(7,73) = 4.77, *p* < 0.001, *R*^2^ = 0.25] and showed a main effect of bully’s gender, (*b* = -0.81, *t*(73) = -2.41, *p* = 0.019, *CI* [-1.47, -0.14]), indicating more perceived threat from the male bully than from the female bully. Moreover, an interaction effect of bully’s gender and NTB, (*b* = 0.20, *t*(73) = -4.55, *p* < 0.001, *CI* [0.11, 0.29]) was found. **Figure 4** shows that for the female bully, the perception of bullying increased with the NTB score, while the opposite pattern applied for the male bully.

There was no effect of the need to belong on the reported mental state or on the intention to formally report the bullying, and neither of the moderators influenced the effects of the independent variables.

Although the overall model for informal report was also not significant, *F*(7,73) = 1.68, *p* = 0.129, *R*^2^ = 0.19, a main effect of NTB emerged (*b* = 0.75, *t*(73) = 2.32, *p* = 0.023, *CI* [0.01, 0.14]), suggesting that the higher the NTB, the greater the likelihood of an informal report.

### Neuroticism

**FIGURE 5:**
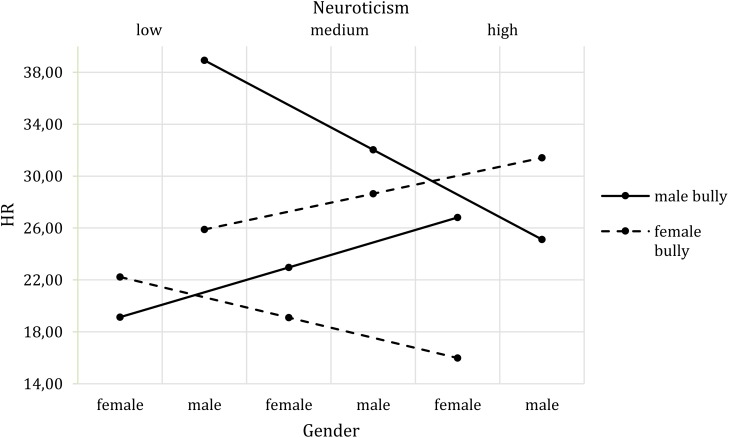
Three-way interaction effect of Bully’s Gender^∗^Participants’ Gender^∗^Neuroticism on HR.

To examine the impact of neuroticism, we conducted the corresponding moderation analyses. There was no influence of neuroticism on skin conductance level. With regard to heart rate, the same participant gender effect as in H1 emerged. In addition, a three-way interaction effect of bully’s gender, participants’ gender and neuroticism (*b* = 27.56, *t*(73) = 2.17, *p* = 0.033, *CI* [2.25, 52.86]) on heart rate was found. **Figure 5** depicts the interaction effect for low, medium and high levels of neuroticism. The Johnson-Neyman technique further indicated that the interaction effect of bully’s gender and participants’ gender on heart rate changed significantly at neuroticism values above 0.93 (9.88%). Bullies of both genders elicited an increase in HR in participants with high neuroticism scores.

**FIGURE 6:**
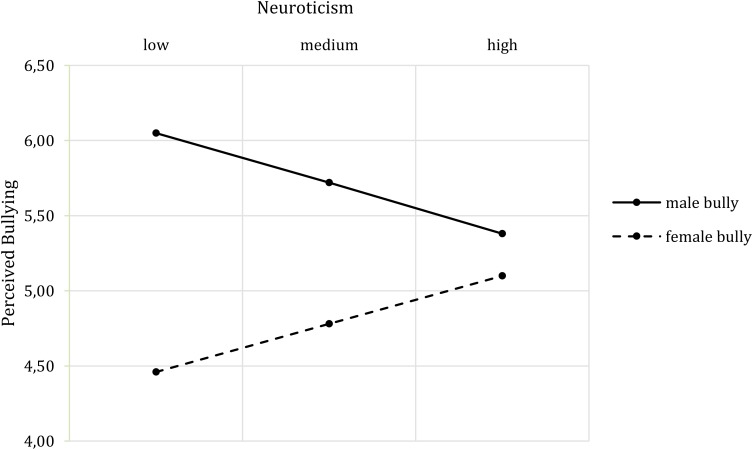
Two-way interaction effect of Bully’s Gender^∗^ Neuroticism on perceived bullying.

The overall model on bullying perceptions was not significant, *F*(7,73) = 2.01, *p* = 0.065, *R*^2^ = 0.15, but showed the same main effect of bully’s gender on bullying perception as in H2. However, a two-way interaction effect of bully’s gender and neuroticism (*b* = 1.20, *t*(73) = 2.08, *p* = 0.041, *CI* [0.05, 2.35]) emerged. **Figure 6** shows that with increasing neuroticism, the perception of bullying by the male bully decreased while the perception of bullying by the female bully increased.

**FIGURE 7:**
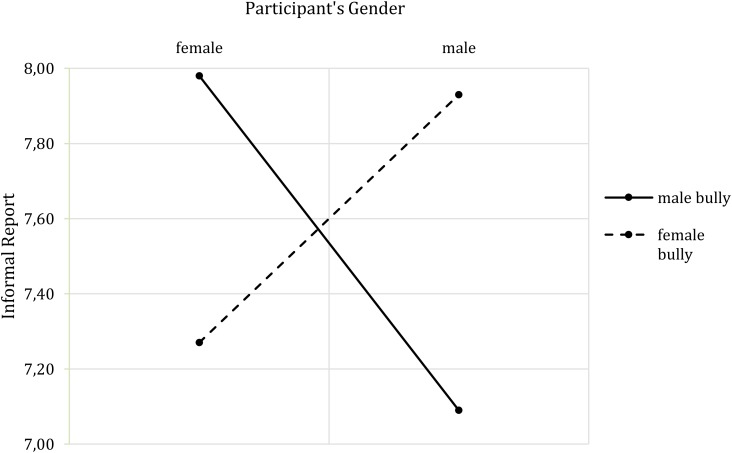
Two-way interaction effect of Bully’s Gender^∗^Participants’ Gender on informal report.

**FIGURE 8:**
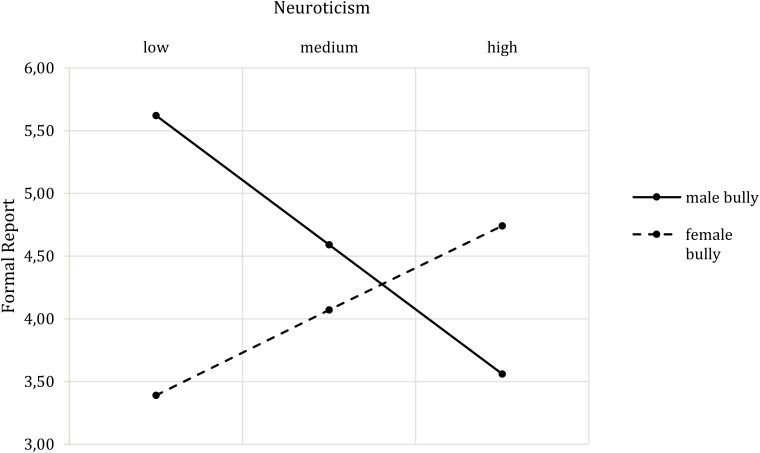
Two-way interaction effect of Bully’s Gender^∗^Neuroticism on formal report.

Concerning the reported mental state, the overall model was not significant and did not reveal any significant main or interaction effects. Regarding the behavioral intentions, the overall model of informal report was not significant *F* (7,73) = 1.39, *p* = 0.223, *R*^2^ = 0.07, while the model of formal report was significant *F*(7,73) = 2.44, *p* = 0.026, *R*^2^ = 0.14. The informal model nevertheless revealed a significant interaction of bully’s gender and participants’ gender (*b* = 1.56, *t*(73) = 2.14, *p* = 0.035, *CI* [0.11, 3.01]), which was not present when neuroticism was excluded from the model. Here, a cross-gender effect emerged: Female participants would be more likely to report mistreatment by a male bully than by a female bully, while the opposite was the case for male participants (**Figure 7**). The analysis of formal reporting of the bullying situation showed an interaction of bully’s gender and neuroticism (*b* = 2.83, *t*(73) = 2.97, *p* = 0.004, *CI* [0.93, 4.73]). **Figure 8** shows that with increasing neuroticism values, participants would be more likely to report the female bully. The opposite pattern emerged with a respect to the male bully: The lower the neuroticism, the greater the likelihood of reporting mistreatment.

### Social Gender

To test whether participants’ social gender influences their experiences and reactions, we calculated models with self-reported masculinity and femininity.

Masculinity did not show a distinct influence on skin conductance level or heart rate. Concerning perception of bullying and mental state, the inclusion of masculinity did not change the results reported in H1–H3. Only the behavioral intentions to either formally or informally report the mistreatment were partially affected by masculinity. The overall model for formal report was significant, *F*(7,73) = 2.20, *p* = 0.044, *R*^2^ = 0.12, and revealed a significant effect of masculinity (*b* = 1.14, *t*(73) = 2.18, *p* = 0.032, *CI* [0.10, 2.19]): Masculinity was positively correlated with the probability of formal report. The model on informal report was not significant, *F*(7,73) = 1.20, *p* = 0.314, *R*^2^ = 0.07.

**FIGURE 9:**
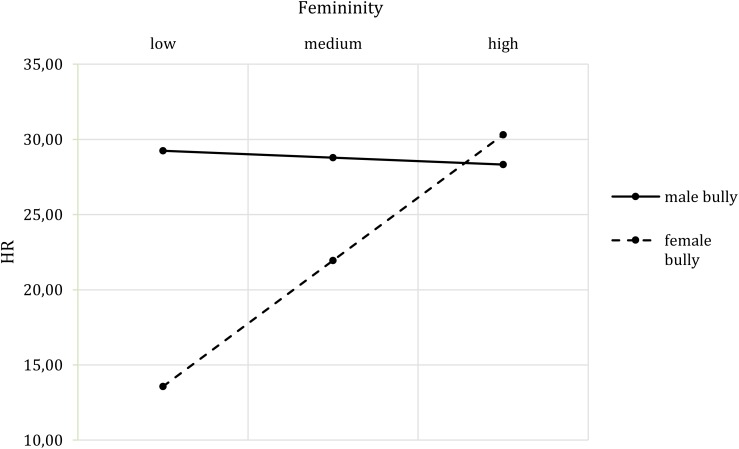
Two-way interaction effect of Bully’s Gender^∗^Participants’ Gender^∗^ Femininity on HR.

The inclusion of femininity did not lead to different results with regard to skin conductance and heart rate. Additionally, a two way interaction of bully’s gender and femininity (*b* = 19.38, *t*(73) = 2.30, *p* = 0.024, *CI* [2.58, 36.18]) emerged. With increasing scores on femininity, the heart rate increased in response to the female bully, while femininity did not affect the heart rate in response to the male bully (**Figure 9**). There were no effects on either of the self-reports or the behavioral intention variables.

In sum, the results indicate that with the exception of heart rate, which was higher for men than for women, women and men react similarly to a bullying scenario in a virtual environment (H1). H2 shows that regarding the gender of the bully, the male character was perceived as more threatening. There was no interaction between participants’ and bully’s gender. With regard to potential moderators, only self-esteem did not prove to be influential, while social gender, neuroticism and need to belong showed various interactions, which are discussed in greater detail below.

## Discussion

The aim of the current study was twofold: We (a) employed virtual environment technology as a testbed in order to learn more about the influence of a bully’s gender and of participants’ resilience factors on the effects of bullying, and (b) evaluated the effects of the gender of the bully (male vs. female) in one bullying situation (acting rehearsal) on the stress level, self-reported mental state, and behavioral intentions of two groups of participants (men and women). This should form the basis for an effective training intervention which might be applied as a training environment in prevention workshops in schools or universities. Therefore, we conducted an experimental between-subjects study in which we varied the bully’s and participants’ gender. A virtual environment was used to create a mild bullying situation by a figure of authority and captured participants’ (adverse) reactions by means of physiological data, self-reports and behavioral intentions to report the mistreatment. Contrary to expectation (H1), we did not find that female victims experience more adverse reactions during a bullying situation than male victims. On the contrary, males experienced a stronger increase in heart rates than did females. In line with Fowles (1980), this can be interpreted as an increased action readiness, and might be an indication that men tend to react more physically to threat. However, this needs to be further investigated in future studies. Despite this one difference, it seems that taken individually, the reactions of female and male victims are very similar. Moreover, moderators hardly changed these results. For potential applications of the virtual environment, this means that it might be useful to provide resilience training to both women and men.

We further assumed (H2) that a male bully would elicit more adverse reactions in participants, due to stereotypical beliefs about men and their different physical appearance. While we did not find any main effects for the physiological measures, the analyses revealed that the evaluation of the bullying situation is indeed more negative when the bully is male. Although participants did not report feeling worse, they described the situation as more threatening. While this might seem unsurprising at first glance, it is nevertheless remarkable that the same behavior leads to different effects if only the gender of the bully is changed. The fact that the same behavior displayed by women and men does not necessarily lead to the same effects or attributions has already been demonstrated in other realms (Deutsch et al., 1987). Although the male and the female bully’s behavior were experienced differently, behavioral intentions to report the mistreatment were not affected by the bully’s gender. For potential future application in resilience training, the more menacing effect of the male bully nevertheless suggests that to increase the effectiveness of such training, it may be more beneficial to include a male rather than a female bully. In addition, the gender could be customized to the “victim’s” preferred degree of experienced threat.

According to H3, we expected that the male bully would trigger the strongest adverse reactions in female victims, due to the above-mentioned reasons. In contrast, we supposed that the female bully would elicit less adverse reactions especially when interacting with male victims. The analyses did not reveal any significant interaction effect of the bully’s gender and participants’ gender, which is in line with the results regarding the main effects of bully’s and victims’ gender, suggesting that overall, gender is rather unimportant concerning the effects of bullying.

However, the consideration of further moderators changes the influence of the bully’s gender on different adverse reactions and the interaction of bully’s gender and participants’ gender. We considered neuroticism, need to belong, self-esteem and social gender as potential moderators. Surprisingly, the only moderator that did not influence the results was self-esteem. This was particularly unexpected given that previous research (Sapouna and Wolke, 2013) indicated that high self-esteem would enable victims to cope better with such a situation. Our results indicate that high self-esteem did not lead participants to evaluate the situation as less threatening or to feel better. However, as we focused on the immediate reaction in the situation, this does not preclude that long-term coping might be more successful in participants with high self-esteem.

The impact of the bully’s gender on the adverse reactions was affected by neuroticism, the need to belong and social gender. Starting with neuroticism, the results indicate that the higher the participants’ neuroticism scores, the more they perceived the female bully as threatening. The opposite pattern was observed for the male bully. While participants with low neuroticism found female bullies less threatening than males, people with high neuroticism evaluated both genders to be equally threatening. Moreover, participants with high neuroticism scores were more likely to formally report mistreatment from a female bully than from a male bully. Male bullies elicit more threat, while female bullies are perceived as less threatening, which may lead to less fear of complaining about mistreatment by females. Moreover, as female bullies are acting against their perceived female role of being warm and sincere, this violation may elicit a desire to penalize them (Bosson and Michniewicz, 2013), in this context through a formal report.

Moreover, the need to belong (Baumeister and Leary, 1995) moderated the relation between the bully’s gender and the perception of the bullying situation. The higher the participants’ need to belong scores, the more threatened they felt by the female bully, while the male bully induced less threat. We assume that these findings are also attributable to gender stereotypes. Participants with a high need to belong, who have a strong wish for attachments and acceptance, might believe that it is easier to befriend females, as females are expected to be friendlier and more communicative and approachable (Eagly and Karau, 2002). However, in the case of the present scenario, the female bully violated her gender role, which in turn might be perceived as particularly threatening. In contrast to this, males are perceived as less approachable, more dominant and less communicative (Prentice and Carranza, 2002); thus, the male bully was acting more in accordance with his perceived role as a male. Another indication that the female bully was perceived as a role-violating person is provided by the interaction effect of the bully’s gender and self-reported femininity on increase in heart rate. The more feminine attributes participants hold, the higher the heart rate increases when encountering the female bully, while the heart rate in response to the male bully was unaffected. One might assume that participants who indicate being more sensitive might more easily notice such a role violation, resulting in a higher heart rate, although this assumption is highly speculative at this point. The fact that only a physiological measure was affected, which is hard to control, might indicate that stereotypical beliefs are embedded on an implicit level, but controlled on an explicit level (i.e., self-report on perceived bullying). However, it needs to be acknowledged that the results on the other psychophysiological variable, skin conductance level, did not manifest themselves in exactly the same way. Although it might be seen as troubling that the two physiological measures did not yield the same results, such findings were also demonstrated in recent studies employing first-person shooter games (Drachen et al., 2010). A potential explanation for the differing impact on different psychophysiological measures might lie in the distinction between the behavioral activation system (BAS) and the behavioral inhibition system (BIS) (Fowles, 1980): While the BAS initiates behavior (approach) and is strongly associated with heart rate, the BIS is an anxiety system, which inhibits behavior and is associated with electrodermal activity. Against this background, a uniform reaction of heart rate and electrodermal activity would not be expected.

Besides the aforementioned effects, neuroticism and the need to belong also affected the interaction between bully’s gender and participants’ gender regarding physiological responses and behavioral intentions. All of these moderations show the same pattern, which indicates specific cross-gender effects. High neuroticism values affected the relation between bully’s gender and participants’ gender with respect to heart rate and informal report. Highly neurotic female participants showed larger heart rate changes in response to a male bully than to a female bully. The opposite was the case for highly neurotic male participants (note, however, that the highest increase in heart rate was observed in men with low neuroticism scores being bullied by a male, which is not in line with the pattern described). When only the cross-gender effects are addressed, it seems that in line with the construct of neuroticism (Eysenck, 1947), highly neurotic persons experience high stress levels especially when they are bullied by a person of the opposite sex. While research has shown that males more often bully males and that both females and males mistreat females (Narayanan and Betts, 2014), it might be that the unusual situation of male participants being oppressed by a female bully led the heart rate to increase. In contrast, although females might have experience of being bullied by both genders, the physical appearance of the male bully might have been more intimidating, leading to the increased heart rate. Although this claim cannot be corroborated by previous research, it seems generally plausible to assume that male bodies are perceived as more threatening. However, would this matter in a VR environment, in which no physical harm can be done? This also needs to be addressed in future research, but for the moment, in line with media equation assumptions (Reeves and Nass, 1996; Krämer et al., 2015), we suggest that people automatically react to virtual characters in the way they would toward real humans.

The same pattern of results was revealed for the intention to informally report the mistreatment: Highly neurotic males would be more likely to report bullying by a female to their friends, and might be disturbed by the role-incoherence of the female bully. In turn, highly neurotic females would be more likely to report bullying by a male to their friends.

The analyses showed a further interaction effect of participants’ gender and bully’s gender on skin conductance for participants with a very low and a very high need to belong. Participants with a low need to belong had very similar skin conductance levels in response to the male and female bully, with the notable exception that male participants reacted more strongly to the female oppressor. However, those participants with a high need to belong seem to react in a special way to a same-sex bully: Female participants showed an increase in skin conductance level in the presence of the female bully, while male participants showed such an increase in response to the male bully. Female participants were rather unaffected by their need to belong level in the presence of a male bully; indeed, those with a high need to belong even showed a slight decrease in their skin conductance level. Most notably, the combination of a male participant being oppressed by a female bully was strongly affected by the participants’ need to belong: While male participants with a low need to belong had a very strong skin conductance increase, the conductance was rather low in those with a high need to belong. This pattern is – as is customary with three-way interactions – very difficult to interpret, but seems to indicate that especially for male participants, the need to belong influences their reactions, rendering male participants with a low need to belong especially susceptible to the female bully. Moreover, the results indicate again that people with a high need to belong rather strive for same-sex connections and are more affected when they are bullied by their own sex. With regard to psychophysiological reactions, however, this pattern only emerged for skin conductance. This might indicate that in this regard, reactions are less connected to energizing activity, and are rather associated with inhibition and anxiety (Fowles, 1980). Given that we cannot exhaustively explain the patterns (e.g., why people even feel threatened when they have a low need to belong, which appears to suggest that the need to belong is not a prerequisite for reactions), future research needs to incorporate the need to belong.

It is also very important to take a closer look at what the results might mean for the identification of resilience factors: In line with results by Friborg et al. (2005), neuroticism affected the outcomes and especially influenced the impact of the bully’s gender. However, low neuroticism or emotional stability did not ease reactions in general, but only depending on the bully’s gender. Therefore, it cannot be seen as a general resilience factor. Findings regarding the role of need to belong were also mixed. While this trait affected perceived bullying and skin conductance, the results did not reveal a clear pattern. At the very least, this construct is worthy of inclusion and testing in future studies. Concerning the question of whether social gender can serve as a resilience factor, we found one single effect of self-reported masculinity on the willingness to formally report the bullying, indicating that with increasingly masculine attributes, the likelihood of reporting the mistreatment in a formal way increased. Given that masculine attributes comprise self-confidence and the ability to deal with pressure (Stein et al., 1992), it is logical that these attributes would support the participant to defend her/himself by reporting the mistreatment in formal situations. This is in line with findings that self-esteem fosters resilience (Sapouna and Wolke, 2013), although this effect did not directly emerge in our study. Moreover, social gender influenced the effect of the bully’s gender on heart rate changes. In conclusion, we recommend the inclusion of social gender in further studies. However, as the reliability of the scales used in our study proved to be rather low, we would suggest the use of different instruments. Furthermore, an explicit consideration of androgyny would be beneficial, as this could turn out to be a more adequate resilience factor than masculinity, the effects of which were rather ambiguous.

With regard to the overarching question of whether the system could be used to train resilience and appropriate behavior after a bullying event, we can conclude that future developments in this direction would be worthwhile. As the results show that people react to bullies in virtual environments, and feel especially threatened by a male bully, the environment could conceivably be used to train resilience against bullying. The experience might, for example, be integrated into a workshop, in which participants learn to withstand the bullying, regulate their emotions, and are taught appropriate responses – to the bully as well as regarding the reporting of the behavior.

The study is, of course, not without limitations. Most importantly, our results are limited to showing the effects of bullies of different gender on participants of different gender, additionally considering the role of several person variables. Our design cannot provide insights into the general question of whether the effects in the virtual environment differ from those in the real world and/or whether the effects are due to the specific bullying rather than the acting rehearsal situation. Although questions such as these have been targeted in previous research, it might be useful to address them again in further studies that include the appropriate control groups.

Although the sample is of a reasonable size for a laboratory study, the number of participants is nevertheless rather low when considering three-way interactions. Furthermore, the sample was quite homogenous and included mostly students. Therefore, the results are only generalizable to students – although students constitute one of the most important target groups for future training interventions. Given the specific setting we used (bullying by a figure of authority in an institutional setting), the results might also not be generalizable to other, more informal bullying by peers. However, as a first step, we aimed to gain insights into people’s willingness to report misbehavior of a superior, as this might even be more difficult and worthy of training compared to reporting bullying by peers. The specific setting also entailed a situation that might not have been appealing to all participants, although this would likely have been true for any type of task. However, it should be noted here that the situation might not have been sufficiently threatening, as the mean values show only mild perceptions of threat. Moreover, the setting only included a small amount of ridiculing, although this is a frequent element of bullying. Another potentially problematic aspect of the virtual environment is the fact that the second virtual character, the fellow student, was always male. Although this was kept constant in all conditions, it might have influenced especially female participants in specific ways.

Some limitations regarding the dependent variables also need to be noted. For example, the mental state scale was developed as a scale for clinical samples, which always bears the risk that there is only limited variance in a sample with non-clinical participants. However, variance appeared to be in a normal range in the present study. The psychophysiological measures have to be treated with caution, as the Empatica E4 has not been validated in previous studies, meaning that it is unclear whether the data might be influenced by artifacts. Another methodological problem is that during the baseline measurement, participants already knew whether they would be interacting with a female or male instructor, since they had seen a picture of the virtual character in the instructions. This might have attenuated the effects. For future studies including gender (especially gender of the bully), it would be advisable to collect data on stereotypical beliefs. An awareness of the participants’ gender stereotypes might facilitate the interpretation of some of the results. With regard to the person variables and potential moderators, we included those which have already been described in the literature (such as neuroticism, gender and self-esteem), but other variables, for instance prior experience with verbal and physical bullying, might, of course, also have influenced the results.

## Conclusion

The present study demonstrates that a virtual bullying situation can have distinct effects. With regard to basic research, we conclude that the use of such an environment enables researchers to deepen their understanding of processes in bullying situations and to identify factors that influence victims’ reactions and resilience. Specifically, the results demonstrate that a male bully is perceived to be more threatening than a female bully, and that men in particular react to bullying with an increased heart rate, which might indicate their readiness to act. Moreover, the personality factors neuroticism, need to belong and social gender moderate the results. With a view to future applications, the environment could indeed be used in order to prepare people for potential future bullying situations – especially when a male bully is used. The experience might be used as part of an education program that builds on the emotional reactions by reflecting on appropriate reactions and training self-regulation of one’s own emotions, as well as learning about appropriate further actions such as formal reporting.

## Ethics Statement

This study was carried out in accordance with the recommendations of the APA with written informed consent from all subjects. All subjects gave written informed consent in accordance with the Declaration of Helsinki. The protocol was approved by the local ethics committee of the Department of Computer Science and Applied Cognitive Science of the University of Duisburg-Essen.

## Author Contributions

Conceptualization of the study: NK, SM, and DF. Development of the virtual environment: SM and DF. Collection of the data: ET. Technical support for data collection: DF. Data analyses: ET and SS. Writing of the manuscript: NK and SS. Editing of the manuscript: SM.

## Conflict of Interest Statement

The authors declare that the research was conducted in the absence of any commercial or financial relationships that could be construed as a potential conflict of interest.

## Supplementary Material

VIDEO 1 Oculus video of the bullying situation featuring the male perpetrator.
